# Dynamics of hospitalizations and in-hospital deaths from COVID-19 in northeast Brazil: a retrospective analysis based on the circulation of SARS-CoV-2 variants and vaccination coverage

**DOI:** 10.4178/epih.e2022036

**Published:** 2022-04-05

**Authors:** Paulo Ricardo Martins-Filho, Adriano Antunes de Souza Araújo, Lucindo José Quintans-Júnior, Bárbara dos Santos Soares, Waneska de Souza Barboza, Taise Ferreira Cavalcante, Victor Santana Santos

**Affiliations:** 1 Federal University of Sergipe, Sergipe, Brazil; 2 Municipal Health Department, Aracaju City Hall, Sergipe, Brazil; 3 Federal University of Alagoas, Alagoas, Brazil

**Keywords:** COVID-19, SARS-CoV-2, SARS-CoV-2 variants, COVID-19 vaccines

## Abstract

This study investigated the dynamics of hospitalizations and in-hospital deaths from coronavirus disease 2019 (COVID-19) throughout the pandemic in northeast Brazil, the Brazilian region with the worst socioeconomic indicators. In total, 141,445 cases, 8,213 hospital admissions, and 1,644 in-hospital deaths from COVID-19 were registered from March 14, 2020 to February 5, 2022. The overall rates of hospitalization and in-hospital deaths were 5.8% and 20.0%, respectively. The hospitalization and death rates significantly decreased over time, which may have been related to progress in vaccination. During the spread of the Gamma variant (January to June 2021), most hospitalized individuals were young adults, and approximately 40% of deaths occurred in this age group. During the predominance of Delta (July to December 2021), over 75% of deaths occurred among the elderly and unvaccinated or partially vaccinated individuals. This rate decreased to 42.3% during the transmission of the Omicron variant (January to February 2022), during which 34.6% of deaths were recorded among fully vaccinated individuals (2 doses) and 23.1% among those who received full vaccination and a booster. The Omicron-driven third wave was associated with a rise in the proportion of deaths among vaccinated individuals, especially among those who had not received a booster dose.

## INTRODUCTION

Coronavirus disease 2019 (COVID-19) is a primarily respiratory disease caused by severe acute respiratory syndrome coronavirus 2 (SARS-CoV-2), with higher mortality rates in male, older people, and those with pre-existing clinical conditions, including hypertension, diabetes, and heart disease [1]. In addition, it has been shown that the COVID-19 fatality rate is higher in vulnerable populations [2,3]. In Brazil, the lack of a national policy against the disease, the increasing population mobility (including international travels and tourism), the return of face-to-face work activities, difficulties in implementing individual and community measures to mitigate COVID-19 transmission, and delays in vaccination have contributed to the emergence and spread of SARS-CoV-2 variants of concern (VOCs) across the country over time [4].

Although the clinical outcomes of patients with COVID-19 are influenced by biological, social, behavioral, and structural factors, it has also been suggested that the risks of hospitalization, intensive care unit (ICU) admission and mortality are higher among patients infected with SARS-CoV-2 VOCs than among those with the wild-type virus [5]. Moreover, there is evidence of an increased risk of hospitalization and death among unvaccinated individuals [6]. In this study, we investigated the dynamics of hospitalizations and deaths from COVID-19 throughout the pandemic in northeast Brazil, the region with the worst socioeconomic indicators in the country.

## MATERIALS AND METHODS

### Study design

This retrospective cohort study was conducted in the city of Aracaju, the capital of Sergipe State, northeast Brazil, from March 14, 2020 to February 5, 2022. The present study is part of the EpiSERGIPE Project, which aims (1) to monitor the epidemiological behavior of COVID-19 in Sergipe State and (2) to support evidence-informed public health decision-making.

### Context

Aracaju is a coastal city located in the poorest region of the country and has a current population estimated at over 657,000 inhabitants. The first case of COVID-19 in Aracaju was identified on March 14, 2020 in a female patient with recent travel to Spain, while the first death from COVID-19 was confirmed on April 2, 2020. As of February 5, 2022, 141,445 cases (1,671 classified at hospital admission as severe or critical) and 2,461 deaths from COVID-19 were registered by the Aracaju Municipal Health Department. In Sergipe State, the COVID-19 vaccination campaign started on January 19, 2021. On June 30, 2021, the vaccination coverage of the general population was approximately 12%, while for individuals over 60 years of age it was 61.4%. At the time of writing this manuscript, the overall COVID-19 vaccination coverage was 73.1%.

### Eligibility criteria and data collection

All patients residing in Aracaju with COVID-19 confirmed by polymerase chain reaction testing who were consecutively admitted to public and private hospitals in the city were included. Data on COVID-19 were extracted from the microdata catalog and official bulletins of the Aracaju Municipal Health Department. The data collected included (1) confirmed cases, (2) vaccination coverage, (3) hospitalizations (admissions, sex and age distribution, hospitalization rate, bed types [clinical beds and ICU], and length of hospital stay), and (4) in-hospital deaths. We also calculated the hospitalization rate (admissions/confirmed cases), in-hospital mortality rate (deaths/admissions), and death rate by vaccination status (fully vaccinated plus booster dose, fully vaccinated with 2 doses, and unvaccinated or partially vaccinated individuals).

### Statistical analysis

The analyses were stratified into 4 periods of the pandemic according to the predominance of variants in Sergipe State: March to December 2020 (B.1, B.1.1.33, B.1.1.119, B.1.1.28), January to June 2021 (Gamma, Zeta), July to December 2021 (Delta), and January to February 2022 (Omicron). The admission date was used as a reference for the distribution of patients in each period. The hospitalization and death rates according to the periods of the pandemic were compared using the 4-sample test for equality of proportions using the Holm method for p-value adjustment. Differences in the length of hospital stay over time were analyzed using the Kruskal-Wallis test followed by the Dunn post hoc test for multiple comparisons. The p-values less than 0.05 were considered to indicate statistical significance. Analyses were performed using the RStudio (https://www.rstudio.com/) software.

### Ethics statement

The EpiSERGIPE Project was approved by the Ethics Committee of the Federal University of Sergipe (protocol No. 33095120.4.0000.5546).

## RESULTS

During the study period, 141,445 cases of COVID-19 and 8,213 hospital admissions were recorded. The overall hospitalization rate was 5.8%, with significant variations across the different phases of the pandemic (p<0.001). In 2020, the hospitalization rate related to COVID-19 was estimated at 5.9%. From January to June 2021, with the wide circulation of the Gamma and Zeta variants, the hospital admission rate increased to 6.9%. However, with progress in vaccination coverage from the second half of 2021, the hospitalization rates declined to 3.6% and 1.7% during the community transmission of Delta and Omicron variants, respectively. In addition, the length of stay during Omicron circulation was the shortest in the entire series (median, 4.0 days; p<0.001).

Our findings showed that approximately 20% of patients hospitalized with COVID-19 were admitted to the ICU throughout the pandemic, with the highest ICU occupancy rate (27.1%) observed during Delta circulation (p<0.001). No significant differences were observed in hospitalizations according to sex over time (p=0.335). However, we found significant variations in admissions by age (p<0.001). In 2020, we found a high hospitalization rate among children and adolescents (12.1%). Following a period of decline throughout 2021, the hospitalization rate in this age group increased significantly in 2022, especially for children aged 0-4 years. In relation to adults, a different epidemiological pattern was observed. Individuals aged 20-59 years had the highest hospitalization rate (58.0%) from January to June 2021, with the spread of the Gamma and Zeta variants and low vaccination coverage for the general population. Conversely, this was the period with the lowest proportion of admissions among individuals over 60 years of age (37.7%), as more than 60% of this population was already fully vaccinated. However, as of July 2021, the hospitalization rate increased among the elderly and decreased among adults aged 20-59 years as vaccination coverage expanded for this age group.

From March 14, 2020 to February 5, 2022, 1,644 in-hospital deaths from COVID-19 were recorded, and the overall mortality rate was 20%. There was a decline in the mortality rate over time, from 23.2% in the first year of the pandemic to 11.6% in February 2022 (p<0.001). We observed that during Delta circulation, 88.4% of deaths occurred among unvaccinated or partially vaccinated individuals. This rate decreased to 42.3% during Omicron circulation, while 34.6% of deaths were recorded among those fully vaccinated with 2 doses and 23.1% among those fully vaccinated plus a booster dose. The mortality rates according to vaccination status were significantly different between the analyzed periods (p<0.001).

**Figure 1.**
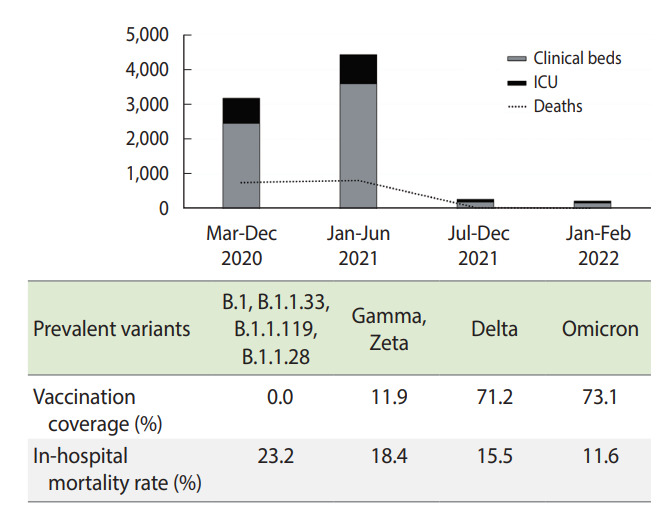
Hospitalizations and in-hospital deaths from coronavirus disease 2019 at different phases of the pandemic according to the circulation of severe acute respiratory syndrome coronavirus 2. ICU, intensive care unit.

Despite a reduction in the proportion of deaths among males, no significant changes were observed over time (p=0.130). During the entire pandemic, at least 65% of deaths occurred among individuals over 60 years of age. The first half of 2021 was the period with the lowest in-hospital mortality rate (60.0%) for this age group (p<0.001). Among adults aged 20-59 years, there was a significant increase in the proportion of deaths from 2020 (25.7%) to the first half of 2021 (39.5%) (p<0.001). In February 2022, the in-hospital mortality rate for this age group was 23.1%. Eleven deaths were recorded among children and adolescents, and the majority (n=7; 63.3%) were in the first year of the pandemic. The dynamics of hospitalizations and deaths from COVID-19 over time are shown in Figure 1. Table 1 presents the details of hospitalizations and deaths according to the circulation of SARS-CoV-2 variants and vaccination coverage.

## DISCUSSION

The overall findings of this study showed that approximately 6% of COVID-19 patients required hospitalization, of which roughly 80% were in clinical beds and approximately 20% were in ICU beds. The in-hospital mortality rate was 20%, with a higher rate observed among males. Although the majority of hospitalized patients were adults aged 20-59 years (about 50%), more than 65% of deaths occurred in individuals over 60 years of age. The results of this study are comparable to those presented in a meta-analysis [7] involving 281,461 individuals with COVID-19 from 11 countries/regions, in which 23% of patients had severe disease, and age and male sex were associated with a higher risk of mortality. However, the ICU bed hospitalizations and mortality rates were lower than those reported in our study, which may be a result of differences in social, economic, and healthcare resources between populations.

Regarding the analyses in specific periods, we observed that during the first wave of COVID-19 in 2020, individuals over 60 years old accounted for the highest proportion of hospitalizations (approximately 46%). Moreover, this period of the pandemic was characterized by the highest in-hospital mortality rate (approximately 23%), concentrated in the oldest age group. The median length of stay was also the longest (8 days) in the entire series, with a rate of admission to ICU beds of approximately 20%. Our results highlight the critical situation of the health system in response to the demand generated by the first wave of COVID-19, especially among the individuals who were most vulnerable to the worst clinical outcomes of the disease. The lack of a national protocol for the care of patients with COVID-19, scientific denialism, overcrowding in hospitals, and the use of personnel without adequate training and experience in the first year of the pandemic may have led to increased mortality, especially in poorer regions.

The second wave of COVID-19 in Brazil, during the first half of 2021, added enormous pressure to the healthcare system with the emergence of the Gamma variant, first detected in Manaus (northern Brazil) in January 2021. A large number of hospitalizations was recorded in a short period, with a significant increase in deaths among adults aged 20-59 years and a decreased mortality rate among the elderly. Although this phase was marked by the beginning of the vaccination campaign, prioritizing health professionals and elderly people, the change in the epidemiological profile of infected patients with SARS-CoV-2 was also observed in other regions of the country [8,9] and may have been associated with the relaxation of restrictive measures and the reopening of trade and services while the country faced a SARS-CoV-2 variant that was roughly 2.5 times more transmissible than that of the first wave [10].

In Brazil, the second half of 2021 was characterized by the spread of the Delta variant, responsible for rapid growth in the number of COVID-19 cases in Europe and the United States in June 2021 [11]. Despite evidence that the Delta variant has potentially faster viral replication and higher infectiousness [12], the number of COVID-19 cases during the period of the prevalence of this variant was 88% lower than that observed during Gamma circulation, which can be explained by the progress of the vaccination campaign in Brazil. In addition, this period had a lower overall hospitalization rate and a reduction of in-hospital deaths compared to the first and second waves. However, there was a high occupancy rate of ICU beds (approximately 27%) among those who needed to be hospitalized and an increase in the in-hospital mortality rate for the elderly, especially among the unvaccinated or partially vaccinated. The Delta variant led to a higher risk of ICU admission than other variants [13], as well as increasing the risk of death in unvaccinated individuals [14].

Although the COVID-19 vaccination campaign in Brazil prevented a third wave associated with the Delta variant, the country experienced a sudden increase in COVID-19 cases in January and February 2022 due to community transmission of the Omicron variant. The Omicron variant was first detected in Brazil in late 2021 in travelers from South Africa; probably due to the relaxation of non-pharmacological containment measures and the lack of systematic surveillance among travelers throughout the country, Omicron-related cases became prevalent across the Brazilian states. In Sergipe State, the first cases were detected in January 2022, which temporally coincided with the final 3 months of our study and may explain the increase in cases in this period. Moreover, we observed a proportional increase in hospitalization rates among children, adolescents, and individuals over 60 years of age, especially in relation to clinical beds. Similar findings were found in the Unite States, where the Omicron variant was associated with less severe outcomes than Delta [15]. During this phase, the median length of stay was 4 days and the mortality rate was approximately 12%, with most deaths occurring among the elderly and unvaccinated or partially vaccinated individuals. In addition, the spread of Omicron was accompanied by an increase in the proportion of deaths among vaccinated individuals, especially among those without a booster dose. There is emerging evidence that the Omicron variant is characterized by immune evasion and that individuals boosted with mRNA vaccines may have increased neutralizing activity against this variant [16,17].

This study showed important changes in the epidemiological profile of hospitalized patients who died from COVID-19 during the pandemic. In addition, the Omicron-driven third wave has been associated with an increase in the proportion of deaths among vaccinated individuals, especially among those who did not receive a booster dose.

## Figures and Tables

**Table 1.** Clinical data and distribution of hospitalizations and deaths of patients with COVID-19 throughout the pandemic

Variables			Mar to Dec 2000	Jan to Jun 2021	Jul to Dec 2021	Jan to Feb 2022
Prevalent SARS-CoV-2 variants			B.1, B.1.1.33, B.1.1.119, B.1.1.28	Gamma, Zeta	Delta	Omicron
Cases of COVID-19 (n)			55,539	64,943	7,654	13,309
Vaccination coverage at the end of the period (%)			0.0	11.9	71.2	73.1
Hospitalizations						
	Admissions (n)		3,253	4,459	277	224
	Sex: male		1,854 (57.0)	2,545 (57.1)	156 (56.3)	114 (50.9)
	Age (yr)					
		0-4	241 (7.4)	124 (2.8)	10 (3.6)	14 (6.3)
		5-19	152 (4.7)	60 (1.3)	4 (1.4)	6 (2.7)
		20-59	1,358 (41.8)	2,586 (58.0)	136 (49.1)	55 (24.6)
		≥ 60	1,499 (46.1)	1,681 (37.7)	127 (45.9)	149 (66.4)
		No information	3 (0.1)	8 (0.2)	-	-
	Hospitalization rate (%)		5.9	6.9	3.6	1.7
	Bed types					
		Clinical beds	2,452 (75.4)	3,588 (80.5)	202 (72.9)	174 (77.7)
		Intensive care unit	721 (22.2)	825 (18.5)	75 (27.1)	50 (22.3)
		No information	80 (2.4)	46 (1.0)	-	-
	Length of hospital stay (day)^1^		8.0 (4.0-16.0)	7.0 (4.0-12.0)	7.0 (4.0-13.3)	4.0 (2.0-7.0)^3^
In-hospital mortality data						
	Deaths (n)		756	819	43	26
	Sex: male		445 (58.9)	458 (55.9)	20 (46.5)	11 (42.3)
	Age (yr)					
		0-4	3 (0.4)	1 (0.1)	0 (0.0)	0 (0.0)
		5-19	4 (0.5)	3 (0.4)	0 (0.0)	0 (0.0)
		20-59	194 (25.7)	324 (39.5)	11 (25.6)	6 (23.1)
		≥60	555 (73.4)	491 (60.0)	32 (74.4)	20 (76.9)
		No information	-	-	-	-
	Mortality rate (%)		23.2	18.4	15.5	11.6
	Death rate by vaccination status (%)^2^					
		Fully vaccinated plus booster dose	-	0.0	2.3	23.1
		Fully vaccinated (2 doses)	-	0.0	9.3	34.6
		Unvaccinated or partially vaccinated	-	100	88.4	42.3

Values are presented as number (%) or median and interquartile range (Q1–Q3). COVID-19, coronavirus disease 2019; SARS-CoV-2, severe acute respiratory syndrome coronavirus 2.

1 Patients discharged.

2 Information retrieved from the database of the Municipal Health Department.

3 Ninety-seven patients remained hospitalized at the time of analysis.
